# Selective Activation of the Wnt-Signaling Pathway as a Novel Therapy for the Treatment of Diabetic Retinopathy and Other Retinal Vascular Diseases

**DOI:** 10.3390/pharmaceutics14112476

**Published:** 2022-11-16

**Authors:** Huy Nguyen, Sung-Jin Lee, Yang Li

**Affiliations:** Surrozen Operating, Inc., 171 Oyster Point Blvd, Suite 400, South San Francisco, CA 94080, USA

**Keywords:** Wnt, diabetic retinopathy, Norrin, FZD_4_, LRP5, therapeutics, BRB, BBB, WNT mimetic

## Abstract

Retinal ischemia, often associated with various disorders such as diabetic retinopathy (DR), retinal vein occlusion, glaucoma, optic neuropathies, stroke, and other retinopathies, is a major cause of visual impairment and blindness worldwide. As proper blood supply to the retina is critical to maintain its high metabolic demand, any impediment to blood flow can lead to a decrease in oxygen supply, resulting in retinal ischemia. In the pathogenesis of DR, including diabetic macular edema (DME), elevated blood glucose leads to blood-retina barrier (BRB) disruptions, vascular leakage, and capillary occlusion and dropouts, causing insufficient delivery of oxygen to the retina, and ultimately resulting in visual impairment. Other potential causes of DR include neuronal dysfunction in the absence of vascular defect, genetic, and environmental factors. The exact disease progression remains unclear and varies from patient to patient. Vascular leakage leading to edema clearly links to visual impairment and remains an important target for therapy. Despite recent advances in the treatment of DME and DR with anti-VEGFs, effective therapies with new mechanisms of action to address current treatment limitations regarding vessel regeneration and reperfusion of ischemic retinal areas are still needed. The Wnt signaling pathway plays a critical role in proper vascular development and maintenance in the retina, and thus provides a novel therapeutic approach for the treatment of diabetic and other retinopathies. In this review, we summarize the potential of this pathway to address treatment gaps with current therapies, its promise as a novel and potentially disease modifying therapy for patients with DR and opportunities in other retinal vascular diseases.

## 1. Introduction

Diabetic retinopathy (DR) remains a leading cause of vison loss in many developed countries and is the most common, specific, and severe microvascular ocular complication of diabetes. According to The International Diabetes Federation, 463 million people were diagnosed with diabetes in 2019 [1]. About one-third of them have some signs of DR and nearly 10% of diabetes patients have vision-threatening DR [2,3]. Chronic hyperglycemia and other causative risk factors such as hypertension are believed to initiate a series of biochemical and pathophysiological changes that ultimately lead to microvascular damage and retinal dysfunction [4,5,6]. In the pathogenesis of DR, including diabetic macular edema (DME), elevated blood glucose can affect the lining of the blood vessels inducing pericyte loss, endothelial cell damage, and basement membrane thickening, which can lead to blood-retina barrier (BRB) disruptions, vascular leakage, and insufficient delivery of oxygen to the retina, and ultimately results in visual impairment [7,8,9]. The vascular leakage and pathologic angiogenesis in the oxygen-deprived retina are often associated with hypoxia-induced overproduction of multiple signaling factors, including vascular endothelial growth factor A (VEGF-A), angiopoeitin-2, angiopoietin-like 4, tissue inhibitor matrix metalloproteinase 1, and hypoxia-inducible factor 1-alpha [10].

Vascular endothelial growth factor (VEGF) is a prominent factor inducing pathological neovascularization and altering retinal capillary permeability [11,12,13]. As a result, anti-VEGF agents such as ranibizumab and aflibercept have been developed to control intraocular vascular abnormalities and are currently considered the standard of care (SOC) in addition to laser photocoagulation in the treatment of DME and wet type age-related macular degeneration (wAMD) [14]. However, real-world data suggest that patients with DME receive fewer anti-VEGF injections in clinical practice compared with randomized clinical trials, resulting in visual acuity outcomes falling short of clinical trial results [15]. Thus, there remains significant need for therapies with improved durability to reduce treatment burden and potentially improve real-world patient outcomes. To that end, considerable efforts are being focused on improving efficacy durability of VEGF pathway inhibitors. For instance, the Port Delivery System with ranibizumab, an intraocular drug delivery system designed for the continuous delivery of ranibizumab (anti-VEGF) into the vitreous for 6 months and beyond (instead of monthly intravitreal [IVT] injection), was recently approved by the Food and Drug Administration (FDA) for the treatment of wAMD and DME [16]. Other pathways beyond VEGF signaling have also been extensively explored. Of these, the Tie2 signaling pathway represents a novel therapeutic target as Faricimab, the first bispecific antibody that targets Ang-2 and VEGF-A designed for intraocular use, has been recently approved by the FDA for the treatment of DME [17] and wAMD [18]. Table 1 includes a partial list of VEGF-binding and non VEGF-binding drugs that are being developed as potential therapies for the treatment of wAMD and DME.

However, as these therapies focus on the vascular leakage and inflammation component of the disease, their impact on vessel regeneration and reperfusion of ischemic retinal areas are minimal. Since vessel regeneration and a reduction of the retinal ischemic area could potentially lead to a durable reduction in hypoxia-induced signaling factors such as VEGF, effective therapies with this mechanism of action would be highly desirable. The Wnt signaling pathway is essential for embryonic development and in adults for tissue homeostasis and injury repair. This pathway has been shown to be critical for proper vascular development and for the maintenance of BRB in the adult retina [28,29]. Dysregulated Wnt signaling has been suggested to contribute to the pathophysiology of DR [30] and modulation of Wnt signaling has been reported to have a beneficial role in various preclinical models. Here, we provide perspective on a new therapeutic strategy that selectively activates the Wnt/β-catenin signaling pathway in retinal endothelial cells as a potential therapy for DR and other retinal vascular diseases.

## 2. Involvement of Wnt/β-Catenin Signaling in Vascular Development and Function

The Wnt signal transduction cascade is a key driver of numerous biological events throughout the life of all animals [31]. The activation of the intracellular signaling cascade initiates when the secreted lipid-modified ligands of the Wnt family bind to surface receptors on target cells. Wnt pathway activation elicits a plethora of cellular responses ranging from cell fate determination, proliferation and migration, body axis patterning during embryonic development to the maintenance of adult tissue stem cells and the regulation of tissue homeostasis and regeneration [32,33]. More in-depth reviews on Wnt signaling can be found elsewhere [32,33,34,35,36,37,38].

**Figure 1:**
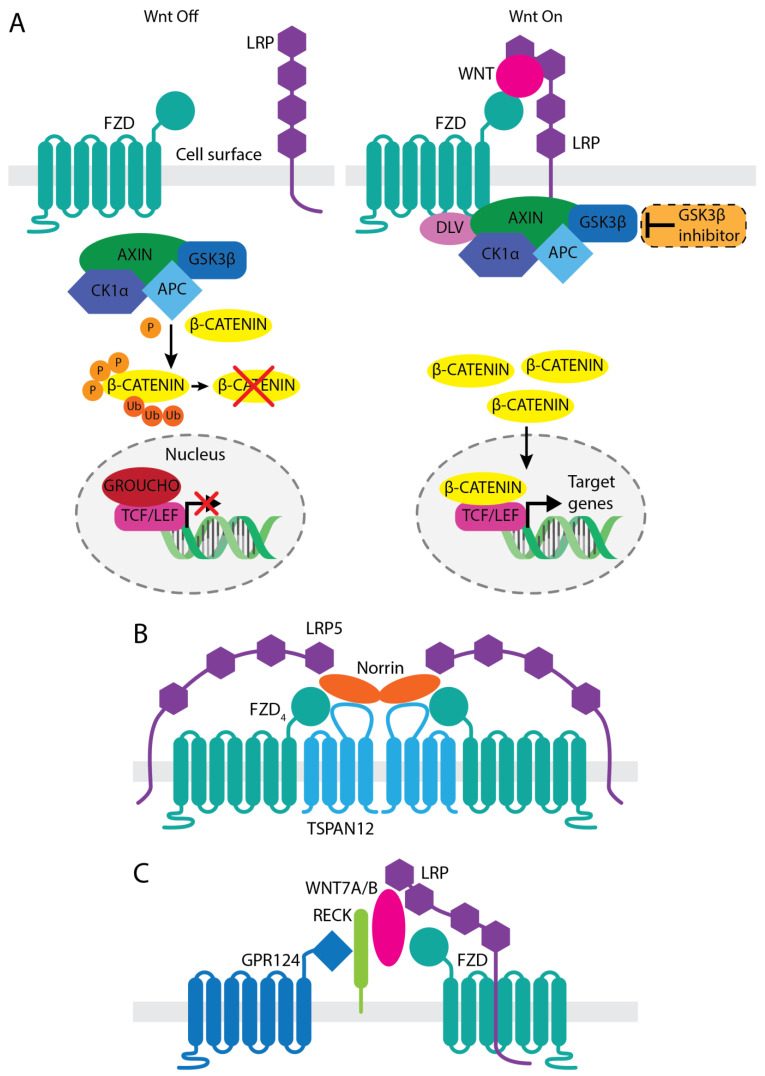
(**A**) WNT/β-catenin signal transduction. (**B**) Norrin signaling complex. (**C**) GPR124-RECK-WNT7A/B signaling complex. The drawings are for illustration purpose only, not drawn to scale nor are the epitopes/binding regions precisely mapped.

The canonical Wnt signaling pathway, referred here as Wnt/β-catenin pathway is the most widely studied Wnt signaling pathway and it involves WNT binding to a heterodimeric receptor complex comprised of a frizzled (FZD) family of 7-pass transmembrane receptors and a single pass transmembrane co-receptor, low-density lipo-protein receptor-related protein (LRP). There are 19 mammalian WNTs, 10 FZDs (FZD_1–10_), and 2 LRPs (LRP5 and LRP6) [32,39]. Following WNT binding, clustering of FZD and LRP results in conformational changes, and subsequent phosphorylation of the receptors. The scaffold protein AXIN, part of the β-catenin destruction complex consisting of adenomatous polyposis coli (APC) along with the serine-threonine kinases casein kinase 1 alpha (CK1α) and glycogen synthase kinase 3 beta (GSK3β), is then sequestered to the cytoplasmic tail of LRP via the FZD-bound disheveled (DVL). As the destruction complex is recruited to the membrane, the transcription factor β-catenin begins to accumulate in the cytoplasm, and subsequently translocates to the nucleus where it binds to T cell factor (TCF)/lymphoid enhancer-binding factor (LEF) transcription factors, inducing downstream transactivation of Wnt target genes (Figure 1A).

Significant evidence has demonstrated essential roles for Wnt/β-catenin signaling in both retinal and central nervous system (CNS) angiogenesis during development and in establishing and maintaining BRB/blood-brain-barrier (BBB) function in adults. In the retina, Norrin, encoded by the *NDP* (Norrie Disease Protein) gene, is secreted by Müller glia and endothelial cells, and plays a critical role in the patterning of the retinal vasculature during development and the establishment of the BRB during adulthood [40,41,42,43]. Norrin, which could be considered an atypical WNT ligand [44,45], is a secreted 131-amino acid protein from the cysteine-knot growth factor superfamily. Norrin forms a dimer that binds with high affinity and specificity to FZD_4_ and LRP5 in the presence of the Norrin-specific co-receptor tetraspanin 12 (TSPAN12), forming a ternary complex that activates the Wnt/β-catenin signal in retinal endothelial cells [46,47] (Figure 1B). *NDP* mutations result in Norrie disease, an X-linked genetic disorder characterized by hypovascularization of the retina, retinal detachment, and severe visual impairments or loss of vision [48,49]. *FZD_4_*, *LRP5*, or *TSPAN12* mutations are also the causes of a spectrum of related congenital retinopathies such as osteoporosis-pseudoglioma syndrome, familial exudative vitreoretinopathy (FEVR), and Coats disease, each of which share resembling phenotypes with Norrie disease. Mutations in *Fzd_4_*, *Lrp5*, *Tspan12*, or *Ndp* in mice result in retinopathies with remarkable similarity to human diseases, including aberrant development of the retinal vasculature, BRB defects, and impaired vision [33,50,51,52,53].

While Wnt signaling mediated through GPR124-RECK-WNT7A/B axis (Figure 1C) is critical for proper CNS angiogenesis and BBB formation, the Norrin/FZD_4_/LRP5/TSPAN12 axis also contributes [47]. Genetic mutant mice of either *Ndp*, *Fzd_4_*, *Lrp5*, or *Tspan12* share phenotypically similar defects in retina and brain vasculature [40,42,46,54]. Knockout (KO) mice show not only retinal vascular hemorrhage, but also BBB breakdown in the cerebellum, which is induced by decreases in endothelial cell tight junction proteins and increases in plasmalemma vesicle associated protein (Plvap) [43,55]. Norrin is also expressed in the inner ear for the maintenance of the stria vascularis, a highly vascularized tissue that produces endolymph in the cochlea [56,57]. Norrin loss of function showed progressive hearing loss with stria vascularis impairment, as seen in Norrie disease patients [56]. Recent research further showed that *Ndp* KO mice have malformations of the microvasculature in the stria vascularis, inducing loss of vessel integrity, resulting in hearing loss [58]. These findings demonstrate the importance of the Norrin/FZD_4_/LRP5/TSPAN12 signaling axis in the development and maintenance of proper functioning vessels in certain organs, including the retina.

Additional roles for Wnt signaling during the development of the retinal vascular system have also been reported. The hyaloid vasculature, a transient embryonic circulatory system that provides oxygen and nutrients to the developing fetal eye, regresses concurrently with the growing and maturing retinal vasculature. This regression of hyaloid vessels occurs immediately after birth in mice and at the fifth month of gestation in humans. Impaired hyaloid regression can lead to an ocular pathology called Persistent Fetal Vasculature (PFV) in humans, which may cause cataracts, glaucoma, intraocular hemorrhages, retinal detachment, and visual impairment [59,60]. Wnt7b is required for hyaloid regression and is expressed in macrophages [61].

Wnt/β-catenin signaling also plays a critical role in the maintenance of barrier function in both the retinal and the CNS vasculature in adults. Acute loss of Fzd_4_ in adult mice results in the loss of barrier function in the retina as well as in the cerebellum and olfactory bulb. Acute induction of Norrin in adult *Ndp* null mice restores cerebellar barrier function. Moreover, constitutively active β-catenin in endothelial cells in the choroid plexus causes upregulation of claudin-5 (Cldn5), a marker normally expressed in an intact BBB, and downregulation of Plvap, a marker of fenestrated BBB [29,62]. Furthermore, Wnt signaling is found to be downregulated in highly permeable capillaries such as the circumventricular organs in the brain and the choriocapillaris as well as the ciliary body and choroid plexus in the retina. Upregulation of Wnt signaling in these permeable endothelial cells causes them to partially convert to a barrier-like state [29].

Given the essential roles of Wnt/β-catenin signaling in the development of the retinal vessels and the maintenance of adult vessel barrier functions, it is not surprising that dysregulated Wnt/β-catenin signaling has been observed in ocular diseases. For example, total β-catenin levels were reported to be elevated in non-proliferative DR patients [63] while the levels of Dickkopf1, an endogenous Wnt signaling inhibitor, was significantly higher in the vitreous of DR patients over that in non-DR groups [64]. Furthermore, both inhibition and activation of this pathway have been reported to have benefits in various disease models [65,66,67,68,69,70]. However, the contribution of Wnt/β-catenin signaling to the pathogenesis of retinal vascular diseases remains to be fully defined.

## 3. Therapeutic Approaches Targeting Wnt Activation

The fact that Norrin/FZD_4_/LRP5/TSPAN12 signaling pathway plays an indispensable role in retinal vascular development and BRB integrity suggests that modulating this pathway may have therapeutic potential to treat retinal vascular diseases. Indeed, overexpression of lens-derived Norrin via gene delivery not only completely restored normal vascular development in Norrin-deficient (*Ndp^y/−^*) mice [71], but it also induced proper vessel regrowth and prevented the pathologic neovascular tuft generation in a mouse retinal ischemic model of oxygen induced retinopathy (OIR) [70]. The potential therapeutic effects in the OIR mice were also observed by treatment with exogenous recombinant Norrin [72]. These results imply that ectopic delivery of Norrin protein or its functional mimetic can have a potential to restore physiological angiogenesis in ischemic retinopathy.

In addition to inducing proper vessel regeneration, Norrin also reduced retinal vascular permeability [43,69,73]. Expressions of key regulators for vascular barrier functions in BRB/BBB such as major facilitator superfamily domain containing 2 (Mfsd2), Cldn5, and zonula occludens-1 (Zo1) are upregulated by FZD_4_ signaling activation, whereas the expression of Plvap is downregulated [29,43,64,68,73], indicating that the FZD_4_ signaling inhibits vascular leakage. In addition, the intravitreal injection of Norrin in streptozotocin-induced diabetic rats partially, but significantly reduced retinal leakage [69]. All these findings demonstrate the effectiveness of Wnt/β-catenin pathway activation in reducing abnormal vascular permeability. The combination of reducing vascular leakage and building normal vessels is an exciting concept that can be tested for retinopathy. Therefore, Norrin/FZD_4_-specific Wnt/β-catenin pathway is a potential target as a new therapeutic strategy for the treatment of retinal vascular diseases including DR.

### 3.1. Anti-LRP5 Antibody That Enhances Norrin Signaling

Although Norrin/FZD_4_/LRP5/TSPAN12 signaling pathway has a significant potential to treat retinal vascular disease, successful pharmacologic FZD_4_ activation through recombinant Norrin for therapeutic BRB modulation and retinal vessel regeneration has remained elusive. Norrin is poorly secreted and is highly associated with the extracellular matrix [74], making it difficult to produce and unsuitable as a therapeutic agent for DR. In addition, Norrin activates Wnt/β-catenin signaling through binding and formation of a ternary complex with receptors FZD_4_, LRP5/6, and TSPAN12 [75,76]. Since many human inherited vitreoretinopathies have mutations in these receptors that affect interactions with Norrin or the assembly of the ternary complex by Norrin, it is unlikely that Norrin itself would be beneficial for individuals harboring these mutations. Therefore, additional approaches will need to be explored to circumvent challenges associated with Norrin and to expand the potential uses to broader retinal vascular diseases.

Activation of Wnt/β-catenin requires the binding of WNT ligands to FZD receptors and to the co-receptors LRP5/6. WNTs bind to the cysteine-rich domain (CRD) on the N-terminus of FZD via two binding sites that have been described as “thumb” (site 1 on FZD) and “index finger” (site 2 on FZD), and the critical palmitoleoyl group on WNT is involved in site 1 binding [77]. LRP5/6 are single-pass transmembrane proteins that contain four tandem YWTD-type β-propeller domains with each followed by an epidermal growth factor (EGF)-like domain, called E1-E4 from N- to C-terminus of the extracellular domain (ECD) [78]. Different WNTs bind different LRP ECD regions with WNT1, 2, 2B, 6, 8A, 9A, 9B, 10B binding to E1E2 and WNT3, 3A binding to E3E4 [78]. It has been reported that bivalent antibodies binding to E1E2 of LRP6 while inhibiting the binding and signaling of E1E2 engaging WNTs, enhance signaling of E3E4-binding WNTs and vice versa [79,80].

**Figure 2:**
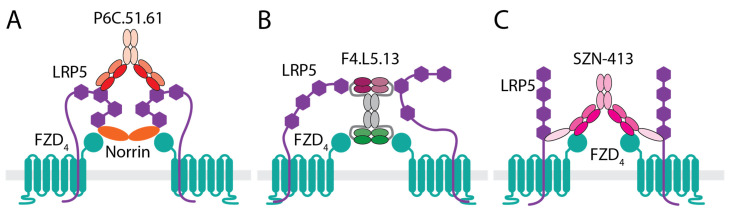
Different approaches to enhance or activate Wnt signaling. (**A**) Anti-LRP5 P6C.51.61 antibody. (**B**) FZD_4_-LRP5 bispecific F4.L5.13 antibody. (**C**) FZD_4_-LRP5 bispecific SZN-413 antibody. The drawings are for illustration purpose only, not drawn to scale nor are the epitopes/binding regions precisely mapped.

Norrin engages FZD_4_ on site 2 and LRP5/6 on the E1E2 domain [75,76]. Similar to what has been observed for WNT ligands, an anti-LRP5 E3E4 antibody, P6C.51.61 (Figure 2A), identified through phage display campaign, enhanced Norrin signaling in HEK293T and human retinal microvascular endothelial cells (HRMEC) in vitro (US10035854). Interestingly, P6C.51.61 also rescued a FZD_4_ mutation, M157V [40] and a Norrin mutation, C95R [46], both identified in FEVR patients that affected either interaction with Norrin or Norrin dimerization, respectively. These results suggest that the clustering of LRP5 by an antibody that binds complementary to Norrin could strengthen ternary ligand/receptor complex formation. Given the critical role of TSPAN12 in Norrin function, mutations in TSPAN12 have been identified in human FEVR patients [81], and *Tspan12* KO mice show stunted retinal vessel development [46]. P6C.51.61 partially rescued the vascular defect and minimally restored formation of capillary network in the retina of *Tspan12* KO mice (US10035854). Treatment with P6C.51.61 in a mouse OIR model also showed trends of reduced pathological neovascular tufts formation and partially induced vascular regrowth in the vaso-obliterated avascular area. These results support the approach of activating Norrin/FZD_4_ signaling for the treatment of retinopathy. However, one of the limitations of this enhancer approach is the reliance on the presence of endogenous ligands. In addition, other Wnt ligands such as *Wnt3a*, *Wnt7a*, and *Wnt10a* are present in the vitreous humor, and some have been reported to be upregulated in OIR [65]. Therefore, it is unclear if the in vivo effects observed with P6C.51.61 are due to its ability to enhance Norrin signaling or to enhance the signaling of these other Wnts present in the vitreous humor that bind Lrp5 E1E2, or even due to inhibition of Wnts that bind Lrp5 E3E4. Therefore, more definitive evidence is still needed to understand whether activation of Wnt signaling in vascular endothelial cells would be beneficial in retinopathy. In addition, a more specific approach may be desirable to reduce potential off-target effects that may complicate data interpretation and therapeutic development.

### 3.2. WNT Mimetics That Activate FZD_4_/LRP5

Another way to enhance Wnt signaling in the retina is through the direct pharmacological activation of FZD_4_/LRP5 mediated Wnt/β-catenin signaling in endothelial cells. Unfortunately, WNT ligands are even more difficult to work with than Norrin due to their lipid modification that makes them insoluble and difficult to express, and their lack of FZD selectivity. However, recent breakthroughs have allowed for the development of FZD-specific WNT-mimetic molecules based on various protein scaffolds [82,83,84,85]. All these approaches utilize bispecific molecules that can simultaneously bind to FZDs and LRPs to induce Wnt/β-catenin signaling. In theory, these approaches can generate molecules with better biophysical properties and with increased receptor specificity that would address the many challenges associated with natural WNTs.

One approach to design WNT mimetics is based on a tetravalent diabody format where two diabodies, each having two binding sites for FZD or LRP, are tethered on two ends of an Fc fragment [83]. Because of this modular design, up to four different binders could be combined into one molecule to achieve the desired receptor and epitope specificity. One WNT mimetic molecule of this format, F4L5.13 (Figure 2B), was constructed by combining a diabody having two binding sites with mono specificity for FZD_4_ with another diabody containing an LRP5 E1 and an LRP5 E3 binder in the tetravalent trispecific configuration [68]. F4L5.13 was reported to specifically activate FZD_4_-mediated Wnt/β-catenin signaling to a similar extent as recombinant Norrin in HEK293 cells. Unlike Norrin, F4L5.13-induced signaling is not TSPAN12-dependent. Treatment of a mouse brain endothelial cell line (bEnd.3 cells) in vitro by F4L5.13 led to rescue of tight junction proteins Zo-1, Cldn3, and Cldn5 expression on the cell surface that was reduced by the pretreatment of these cells with VEGF and led to reduced permeability of the cells in vitro [68]. Since F4L5.13 does not depend on TSPAN12 for signaling, treatment of *Tspan12* KO mice with F4L5.13 restored retinal angiogenesis and barrier function as indicated by increased Cldn5 and decreased Plvap expression. Similar to what was observed with anti-LRP5 Norrin enhancer antibody described above, F4L5.13 treatment also reduced pathological neovascular tufts formation in the mouse OIR model [68]. However, the ischemic area of the retina was not restored by F4L5.13 treatment as no significant effect on the avascular area was observed. It is unclear why F4L5.13 had no impact on avascular area reduction, which was observed by the treatment of the anti-LRP5 E3E4 antibody, P6C.51.61. Perhaps the potency of F4L5.13 needs further improvements as a high vitreous concentration, 500 nM, was necessary to show a significant reduction in neovascularization [68].

Another approach to WNT mimetic generation has focused on other antibody-based formats. Using scFv based fragments, different design parameters, including valency, linker lengths, relative orientation, geometry, stoichiometry, and receptor specificity, were first systematically evaluated [84]. This led to the identification that multivalent binding to FZDs and LRPs was a critical requirement for maximal Wnt/β-catenin signaling with the optimal ratio of two FZD binding arms with one or two LRP binding arms [84]. Subsequently, these design principles were shown to apply to other multivalent antibody formats such as the tetravalent bispecific VHH-IgG format [86], which are highly stable and suitable for large scale manufacturing of drug products. 

**Figure 3:**
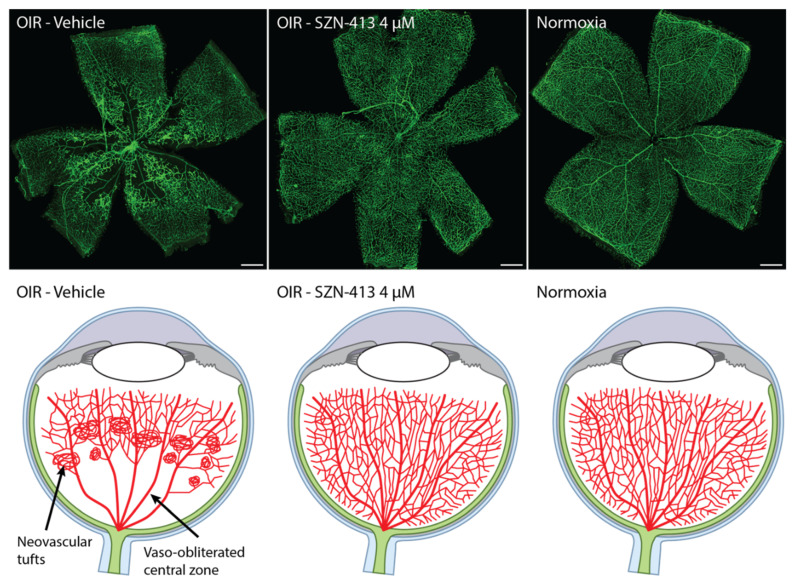
SZN-413 regenerates vessels in the vaso-obliterated central zone and inhibits neovascular tuft formation in OIR retina.

Recently, a FZD_4_/LRP5 specific WNT mimetic molecule, SZN-413 (Figure 2C), was constructed based on the tetravalent bispecific VHH-IgG format [87]. The IgG portion of SZN-413 binds FZD_4_ with high affinity (with K_D_ < pM) and the VHH domains bind LRP5. SZN-413 is highly potent, capable of activating Wnt/β-catenin signaling in vascular endothelial cells such as HRMEC and bEnd.3 cells in vitro and its potency is several orders of magnitude higher than that of recombinant Norrin or WNT3A in vitro. SZN-413 has biophysical and chemical characteristics that translate to desirable solubility, stability, and large scale manufacturability. Similar to F4L5.13, the bispecificity toward FZD_4_/LRP5 may have the advantage of averting promiscuous activation of other FZD family members in the vitreous space as both FZD_4_ and LRP5 are enriched in retinal endothelial cells vs. other cell types [87]. SZN-413 increased CLDN5 and ZO-1 protein levels in HRMEC [87]. The upregulated expression of known BBB/BRB endothelial cell transcripts, LEF1 and MFSD2A [29,73], by SZN-413 further supports that SZN-413 can increase vascular integrity through upregulation of barrier function proteins. In a mouse ischemic retinopathy OIR model, SZN-413 reduced pathological neovascular tufts formation to a higher extent than aflibercept [87]. Furthermore, SZN-413 treatment reduced the size of avascular area significantly more than aflibercept and almost completely restored the normal vessels in the vaso-obliterated central zone in the retina (Figure 3). This implicates that the novel FZD_4_/LRP5 specific WNT mimetic molecule may be able to transform pathologic neovascularization into physiological neovascularization, reducing retinal ischemia and the ensuing overproduction of signaling molecules responsible for the development of pathologic neovascularization in the proliferative stage of diabetic retinopathy. This regeneration of vessels could be disease modifying, and has the potential to have a durable treatment effect; however, this will need to be confirmed in the clinic. In addition, dose–response data have shown that efficacy could be obtained at concentrations as low as 40 nM with a maximum effect at or above 4 µM suggesting that high doses may further extend the durability provided safety is not compromised. More research on the efficacy duration of SZN-413 is needed to determine the dose and delivery frequency of the drug. Even though both SZN-413 and F4L5.13 should be based on a similar mechanism of action the avascular area size reduction was not observed with F4L5.13 in OIR [68]. The reason for this difference is unclear; however, the results may suggest that appropriate molecular format, geometry and potency may be critical to induce appropriate levels of Wnt/β-catenin signaling in a therapeutic setting.

Although inhibition of VEGF-driven retinal leakage is a major consideration for retinopathy treatment, studies to date have not sufficiently provided direct evidence showing WNT ligands can successfully perform this function in vivo. Recently, Diaz-Coranguez et al. showed that intravitreal injection of recombinant Norrin can inhibit the VEGF-driven retinal leakage in rats [69]. Consistent with this finding, Nguyen et al. showed that the SZN-413, a FZD_4_/LRP5 specific WNT mimetic, inhibits the VEGF-driven retinal leakage in rabbit eyes [87]. Moreover, its efficacy was observed at vitreous humor VEGF concentrations >600 µg/mL (1 µg recombinant hVEGF_165_, intravitreally injected into rabbit eye); considering that the VEGF concentration seen in proliferative DR or DME patients is less than 10 ng/mL in vitreous humor [88,89,90], SZN-413 may have therapeutic potential in VEGF-driven retinal leakage diseases such as DR.

### 3.3. GSK-3β Inhibitor

GSK-3β is a key intracellular component of the Wnt/β-catenin signaling pathway. In the absence of WNT ligands, a destruction complex consisting of AXIN/GSK-3β/CK1/APC phosphorylates β-catenin leading to its ubiquitination and degradation. Upon ligand binding to FZD/LRP, the destruction complex containing GSK-3β is recruited to the intracellular side of the receptor complex on the membrane via DVL and AXIN, leading to stabilization of β-catenin and subsequent activation of Wnt target genes [33]. Therefore, inhibition of GSK-3β can lead to stabilization of β-catenin and activation of Wnt/β-catenin signaling (Figure 1A). It was reported that hypoxia increases GSK-3β activity in HRMEC leading to a reduction in β-catenin and its associated impairment in cell/cell junctions, and inhibition of GSK-3β kinase activity by small molecules or peptides improved capillary morphogenesis in a 3D collagen assay in vitro [91]. In a mouse ischemic retinopathy OIR model, GSK-3β inhibitors reduced abnormal vascular tufts, reduced vascular leakage, improved proper re-vascularization, reduced avascular area, and improved retina perfusion similar to what was observed with SZN-413 discussed above [91]. Furthermore, another GSK-3β inhibitor, lithium chloride, also partially rescued the retinal vascular developmental defects in several FEVR models [92,93,94]. A bell-shaped curve was observed both in vitro and in vivo for GSK-3β inhibitors where the beneficial effects were lost at high compound concentrations, which may contribute to the lack of efficacy of some GSK-3β inhibitors in certain studies, suggesting that a careful titration of doses may be necessary to achieve meaningful efficacy for this approach [91,95]. The cause for the bell-shaped dose–response curve is not clear. Therapeutic development for retinopathy based on GSK-3β inhibitors is challenging given the short half-life of these small molecules or peptide inhibitors and the broad impact inhibition of GSK-3 serine threonine kinase activity may have due to its cross talk with many signaling pathways in addition to Wnt [96]. Nonetheless, the reported effects of GSK-3β inhibitors on endothelial cells in vitro and in vivo are consistent with the hypothesis that activation of Wnt/β-catenin signaling could be a novel approach to treat retinal vascular diseases such as DR.

## 4. Conclusions and Future Perspectives

Wnt/β-catenin signaling mediated through the Norrin/FZD_4_/LRP5/TSPAN12 axis has been shown to be essential for the proper formation of retinal vascular structure during development, and for the maintenance of proper vascular structure and function in adults. Activation of this pathway using several different approaches, including the use of Norrin, Norrin enhancing anti-LRP5 antibodies, FZD_4_ activating WNT mimetics, and GSK-3β inhibitors all suggest that activation of Wnt/β-catenin signaling could be a novel strategy for the treatment of retinal vascular diseases such as DR. Common findings from these Wnt activators are an increase in tight junction protein expression, a reduction in vascular leakage in vitro and/or in vivo, and a reduction in pathological neovascular tufts formation in the ischemic mouse OIR model. These findings are consistent with the established role of Wnt/β-catenin signaling in tight junction protein expression and the maintenance of the BRB in adult vessels [29]. In addition, SZN-413 directly suppressed vascular leakage in a rabbit VEGF-induced leakage model [87], suggesting that this mechanism of action can directly counter the effects of VEGF on the integrity of the retinal vessels.

The effects on vessel regeneration are somewhat variable between these Wnt activating approaches. While F4L5.13 rescued the vascular insufficiency in *Tspan12* KO mice, it did not induce vessel regrowth in the ischemic mouse OIR model [68] whereas GSK-3β inhibitors [91] and the Norrin enhancing anti-LRP5 antibody, P6C.51.61 (US10035854) partially reduced the avascular area, and SZN-413 fully regenerated vessels in the vaso-obliterated central zone in the OIR [87]. It is not clear what caused the differences in vessel regeneration observed between the different compounds; it could be possible that specificity and signaling strength may contribute to the differences. Furthermore, clinical studies will be needed to determine if the results observed in the OIR model will translate to humans. Nevertheless, the ability of SZN-413 to fully regenerate vessels in the ischemic OIR model is exciting as it suggests that this mechanism has the potential to address the retinal non-perfusion and ischemia that is not effectively addressed with the current therapies, and therefore could provide a treatment option that can simultaneously address both retinal non-perfusion and vascular leakage in disease. The ability of Wnt/β-catenin signaling to induce proper healing and regeneration has been observed in other tissues as well [86,97,98]. For example, in the intestine, a WNT mimetic not only induced progenitor cell proliferation, but also induced differentiation, setting the tissue onto a normal healing progress, and fully restored the normal architecture of the intestinal structure [98]. The collective observations from the studies summarized here may also suggest a similar effect of the Wnt/β-catenin signaling in the retina where the induction of this signaling pathway (mediated through FZD_4_) shuts down the vicious cycle of vascular break down, hypoxia, production of factors inducing pathological angiogenesis, and restores normal tissue healing process and regenerates physiological vasculatures. It would be exciting to explore whether these healing effects are indeed observed in the clinical setting. This novel mechanism of action of FZD_4_/LRP5 specific WNT mimetic opens new possibilities to treat diabetic and potentially other retinopathies. Although choroidal vessels do not have tight junctions, the observation that activation of Wnt/β-catenin signaling in choroidal endothelial cells reduces permeability [29] offers an intriguing possibility that FZD_4_ activating WNT mimetics may also provide a benefit for wAMD.

FZD_4_ activating WNT mimetics could also have benefits in treating inherited human vitreopathies. Many mutations identified in these patients are located in *TSPAN12*, *FZD_4_*, or *LRP5* that affect Norrin binding or Norrin induced receptor complex formation [99]; therefore, it is unlikely that Norrin treatments would be effective in correcting these genetic defects. As demonstrated by the rescuing of the FZD_4_ M157V and Norrin C95R FEVR mutants by P6C.51.61 and since FZD_4_ activating WNT mimetics do not rely on TSPAN12 to signal and could bind to different regions on FZD_4_ and LRP5 from Norrin, FZD_4_ activating WNT mimetics could effectively induce Wnt/β-catenin signaling in individuals harboring these mutations and offer a potential option for treating these inherited retinal vascular diseases.

While human genetics highlight the importance of FZD_4_/LRP5 in retinal vascular endothelial biology, and expression analysis suggest that FZD_4_/LRP5 may provide more selectivity toward the retinal endothelial cells over other cell types in the vitreous space, other FZDs have been reported to be expressed on retinal endothelial cells [100]. Future research could also explore these other receptors and understand whether there are advantages to target other FZDs beyond FZD_4_. Given the critical role of Wnt/β-catenin on brain endothelial function and maintenance of the BBB, FZD_4_ activating WNT mimetics could find broader applications for conditions characterized by a disrupted BBB such as stroke, epilepsy, and certain neurodegenerative diseases. In conclusion, modulation of Wnt/β-catenin signaling, especially mediated through FZD_4_ activating WNT mimetics, may offer a novel therapeutic approach for retinal vascular diseases such as DR and other various vascular diseases.

## Figures and Tables

**Table 1:** Pipeline drugs in clinical studies for the treatment of wAMD and DME.

Signaling Pathway	Drug	Target	Drug Type	Reference
VEGF-binding	Port Delivery System with ranibizumab	VEGF-A	Monoclonal antibody Fab	[16]
	ADVM-022	VEGF-A, B, Placenta growth factor	AAV-7m8 vector coding aflibercept	[19]
	KSI-301	VEGF-A	Antibody biopolymer conjugate	[20]
	Abicipar pegol	VEGF-A	Small proteins that contain engineered ankyrin repeat domain	[21]
	Faricimab	VEGF-A, Ang-2	Humanized full-length bispecific IgG1 antibody that selectively neutralizes VEGF-A and Ang-2	[17,18]
Non VEGF-binding	AXT107	Integrin	Peptide	[22]
	Nesvacumab	Ang-2	Monoclonal antibody	[23]
	Razuprotafib	Tie2	Small molecule	[24]
	Risuteganib	Integrin	Peptide	[25]
	Anti-LRG1	Leucine-rich alpha-2-glycoprotein 1 (LRG1)	Antibody	[26]
	THR-149	Plasma kallikrein	Peptide	[27]

## Data Availability

Not applicable.
